# Hepatomegaly and type 1 diabetes: a clinical case of Mauriac’s syndrome

**DOI:** 10.1186/s13052-018-0598-2

**Published:** 2019-01-07

**Authors:** Fortunato Lombardo, Stefano Passanisi, Albino Gasbarro, Giovanni Tuccari, Antonio Ieni, Giuseppina Salzano

**Affiliations:** 1 Department of Human Pathology in Adult and Developmental Age “Gaetano Barresi”, University of Messina, Via Consolare Valeria 1, 98125 Messina, Italy; 2 Department of Human Pathology in Adult and Developmental Age “Gaetano Barresi”, Section of Anatomic Pathology, University of Messina, Via Consolare Valeria 1, 98125 Messina, Italy

**Keywords:** Type 1 diabetes, Glycogenosis, Hepatomegaly, Dyslipidaemia, Hyperglycaemia

## Abstract

**Background:**

Hepatic glycogenosis is characterized by excessive glycogen accumulation in hepatocytes and represents a complication of poor controlled type 1 diabetes. It can be caused by excessive insulin doses or recurrent ketoacidosis episodes. Mauriac’s syndrome is a rare disease, which includes short stature, growth maturation delay, dyslipidemia, moon facies, protuberant abdomen, hepatomegaly with transaminase elevation. It has become even less common after the emergence of advances on diabetes treatment, but still exists. Recent reports described glycogenosis without the full spectrum of Mauriac’s syndrome in both adults and children with brittle diabetes. Clinical, laboratory and histological abnormalities are reversible with appropriate glycemic control.

**Case presentation:**

We hereby report a case of 11-year-old male who presented with hepatic glycogenosis mimicking Mauriac’s syndrome. The patient was admitted at our Pediatric Diabetes Clinic for marked hepatomegaly, short stature and for the poor metabolic control. Blood investigations and liver tests excluded most of major causes of hepatopathy. A liver biopsy allowed us to make diagnosis of hepatic glycogenosis. To control hyperglycaemia, initially we titrated daily insulin dosage, and then intravenous insulin treatment was practiced with the consequent normalization of liver enzymes.

**Conclusion:**

Mauriac’s syndrome should be considered in subjects with brittle type 1 diabetes and hepatomegaly.

## Background

Hepatic glycogenosis (HG) is an under recognized condition characterized by pathological storage of glycogen in hepatocytes and represents a rare complication of type 1 diabetes mellitus (T1D) [1]. Glycogen reload in the liver was first described in children by Mauriac in 1930 [2, 3] as a component of Mauriac’s syndrome (MS), a rare disease characterized by hepatomegaly with transaminase elevation, puberty and growth failure, dwarfism, dyslipidaemia, reduction of insulin-like growth factor 1, cushingoid features [4]. MS has been described in children and adolescents with poor insulin compliance and brittle glycaemic control along with inadequate diet [5]. Currently, HG is a disease that occurs at any age and can be present without the full spectrum of features described for MS. In few reports, HG is considered as the primary cause of hepatomegaly in young patients with T1D [6]. High daily insulin dosage and recurrent ketoacidosis episodes significantly increase the risk for HG [7]. In the recent years the occurrence of comorbidities associated to diabetes in children, including HG, is rare due to the intensive insulin treatment in T1D by means of multiple daily injection and continuous subcutaneous insulin infusion, with availability of low cost treatment and wider approach of nutritional programs [8–10]. Nevertheless several paediatric case reports of HG have been reported in literature over the last years (Table 1) [5, 11–22]. The majority of reports demonstrated that an adequate management of glucose and daily insulin dosage could lead to a complete remission of clinical, laboratory and histological abnormalities [23].

**Table 1:** [5, 11–22] Summary of the major paediatric case reports (pub med indexed) on hepatic glycogenosis in type 1 diabetes

Ref.	Age/Sex	Duration of diabetes	Clinical features	Follow-up
Franzese (2001)	14 ys/F	11 years	Hepatomegaly, cushingoid appearance, puberty delay, elevation of aminotransferases and triglycerides	Resolution
Carcione (2003)	3 ys/F	1 month	Hepatomegaly, elevation of aminotransferases	Resolution
Mahesh (2007)	3 ys/M	2 years	Hepatomegaly, short stature, elevation of aminotransferases	Resolution
Aljabri (2011)	13 ys/M	3 years	Hepatomegaly, elevation of aminotransferases	Unknown
Dantuluri (2012)	14 ys/F	5 years	Mild hepatomegaly	Unknown
Lin (2012)	10 ys/F	7 years	Hepatomegaly, elevation of aminotransferases	Resolution
Saisuka (2013)	13 ys/F	4 years	Hepatomegaly, elevation of aminotransferases	Unknown
Gutch (2013)	15 ys/M	8 years	Hepatomegaly, short stature, puberty delay, elevation of aminotransferases	Resolution
Oeschgef (2014)	11 ys/F 10 ys/M 14 ys/F 14 ys/F 13 ys/F	2 years 3 years 11 years 8 years 10 years	Hepatomegaly, cushingoid appearance, elevation of aminotransferases and triglycerides Hepatomegaly, short stature, elevation of aminotrasferases Hepatomegaly, short stature, cushingoid appearance, puberty delay, elevation of aminotransferases Hepatomegaly, short stature, cushingoid appearance, puberty delay, elevation of aminotransferases Hepatomegaly, short stature, cushingoid appearance, puberty delay, elevation of aminotransferases	Unknown Resolution Resolution Poor improvement Resolution
Butts (2014)	13 ys/F	2 years	Hepatomegaly, elevation of aminotransferases	Unknown
Chandel (2017)	12 ys/F	5 years	Hepatomegaly, elevation of aminotransferases	Resolution
Al Sarkhy (2017)	6 ys/F	4 years	Hepatomegaly, elevation of aminotransferases	Resolution
Kocova (2018)	13 ys/M	8 years	Hepatomegaly, short stature, cushingoid appearance, puberty delay, elevation of aminotransferases	Resolution

## Case presentation

We present the clinical case of an eleven-year old boy, born from Romanian non-consanguineous parents who belonged to low socioeconomic strata, affected by T1D since he was 8 years old.

At the onset of diabetes, he was hospitalized in the Emergency Department of a Romanian hospital with a recent history of polyuria, polydipsia, weight loss and weakness. At the admission, the patient presented Glasgow Coma Scale score of 8. The following laboratory test were performed: blood gas analysis showed pH 7.04, bicarbonate serum 6 mmol/l; serum glucose was 567 mg/dl; glycated hemoglobin was 120 mmol/l and ß-hydroxybutyrate levels were 5.6 mmol/l.

He had been treated with insulin therapy, water and salt replacement according to the International Society of Pediatric and Adolescent Diabetes guidelines for management of diabetic ketoacidosis (DKA) for 48 h [24]. After the suspension of DKA treatment, multiple daily insulins injections were prescribed, with an initial total insulin dosage of 1 IU pro kg, insulin lispro at meals and insulin glargine at bedtime. The patient was discharged after one week, but he did not attend follow-up visit at the Diabetes Centre.

**Fig. 1:**
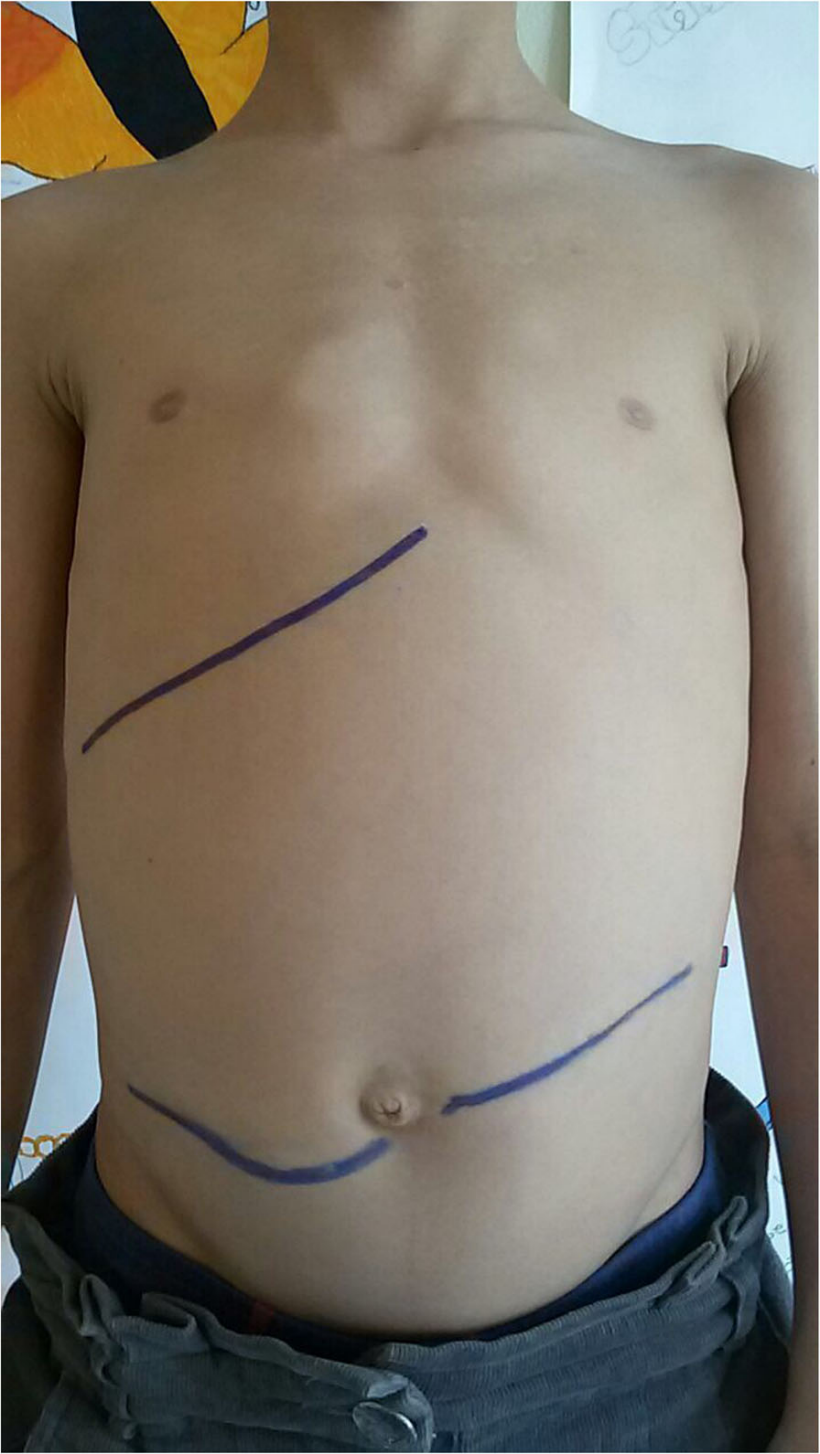
Clinically detected liver boundaries at the admission in our Paediatric Diabetes Centre

The glycometabolic control was very poor and the patient had been hospitalized with moderate diabetic ketoacidosis in two occasions. At the age of 10 years, he had moved to the Southern Italy with his family. At the age of 11 years, he was admitted due to severe DKA in an Emergency Department of a secondary level hospital. After the resolution of the DKA, he was transferred to our Paediatric Diabetes Clinic for further investigations due to the observation of marked hepatomegaly (Fig. 1), short stature and for the poor metabolic control. At the admission, he presented a stature of 127.5 cm and a weight of 25 Kg (< 3° centile of expected height and weight for age and sex). Secondary sexual characters were absent, Tanner stage being 1. On clinical examination, he had a liver enlargement of 4 cm below subcostal margin. No jaundice, splenomegaly, declivous oedema or ascites were noted.

Laboratory tests showed the following alterations: serum glucose (238 mg/dl), glycated haemoglobin (114 mmol/l), total cholesterol (271 mg/dl), triglycerides (175 mg/dl). Acid base balance was normal (pH 7.39, bicarbonate serum 24 mmol/l), lactate serum was 1.1 mmol/l. Liver function tests showed normal levels of transaminases, alkaline phosphatase, total bilirubin and prothrombin time. To evaluate differential diagnosis of hepatomegaly he was submitted to further laboratory investigations. Normal levels of antinuclear antibodies, anti-smooth muscle antibodies, antimitochondrial antibodies and antineutrophil cytoplasmic antibodies excluded autoimmune hepatitis. To rule out infectious causes of hepatomegaly it was found serology for Epstein Barr virus, cytomegalovirus, hepatitis A virus, hepatitis B virus, hepatitis C virus, human immunodeficiency virus, which all resulted negative for recent infections. Normal levels of iron serum studies eliminated the suspicion of hemochromatosis. Normal cupremia and ceruloplasmin levels excluded Wilson disease. To investigate short stature, the following exams were performed: thyroid function tests resulted normal, serologic testing for coeliac disease was negative, insulin-like growth factor 1 was at the lower levels of normality according to age and sex. The skeletal age determination showed 9.9 years Greulich-Pyle atlas. Clonidine growth hormone stimulation test was performed and revealed subnormal growth-hormone peak level (6.9 ng/dl).

Abdominal ultrasound confirmed marked hepatomegaly with regular echo texture and normal portal vein. During the hospitalization, he presented a brittle glycaemic control characterized by fluctuations between hyperglycaemia and hypoglycaemia. In order to obtain a good metabolic control, the daily insulin dosage was titrated reaching a daily insulin dose of 2.3 IU pro kg. His parents received an education diabetes program. MS was hypothesized based on the association of hepatomegaly, short stature, dyslipidaemia and a history of poorly controlled diabetes.

**Fig. 2:**
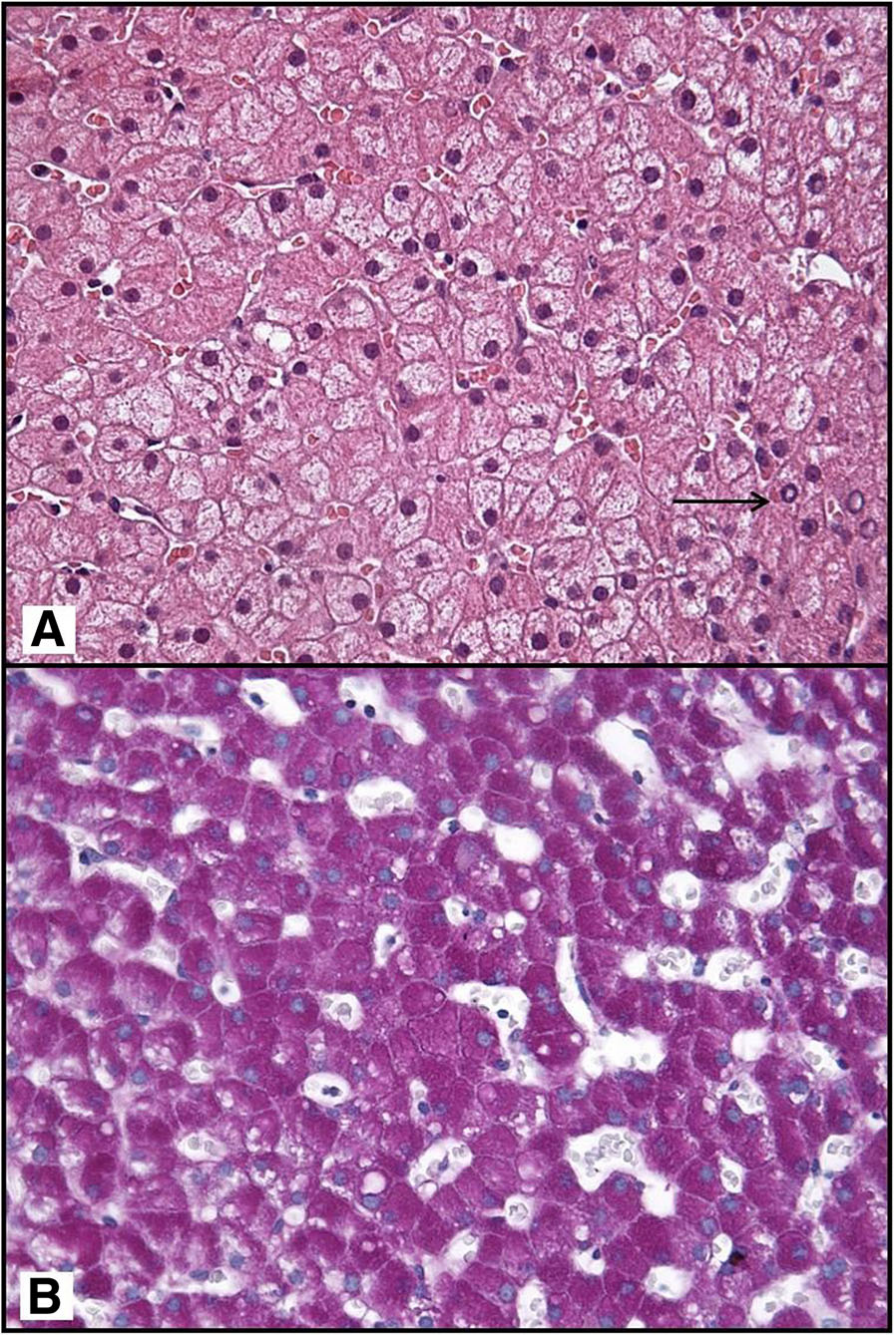
Histological features of liver glycogen reload. The liver biopsy showed normal architecture with diffuse hepatocellular changes characterized by swollen hepatocytes with pale, clear-staining cytoplasm. Few glycogenated nuclei are present in periportal hepatocytes (arrow) (**a**, Haematoxylin/eosin stain, X360). A large amount of glycogen deposits revealed by PAS stains (**b**, nuclear Mayer’s Haemalum counterstain, X360)

Liver biopsy was performed, routinely haematoxylin-eosin stained 4 μ-thick sections were made from 10% neutral-buffered formalin-fixed paraffin-embedded tissue block. Parallel serial sections were also stained with periodic acid-Schiff, Sirius Red, Orcein, Perls and Masson’s trichromatic techniques. The sample showed a preserved lobular architecture with many swollen glycogen-laden hepatocytes, prominent periportal nuclear glycogen pseudo-inclusions (Fig. 2a) and focal macrovescicular steatosis (< 33%). Staining with periodic-acid Schiff showed an intense cytoplasmic positivity, with a strong magenta’s colour in swollen hepatic elements (Fig. 2b). No evidence of inflammation and fibrosis was noted. Staining for copper and iron deposits were negative. These findings confirmed the diagnosis of hepatic glycogenosis.

**Fig. 3:**
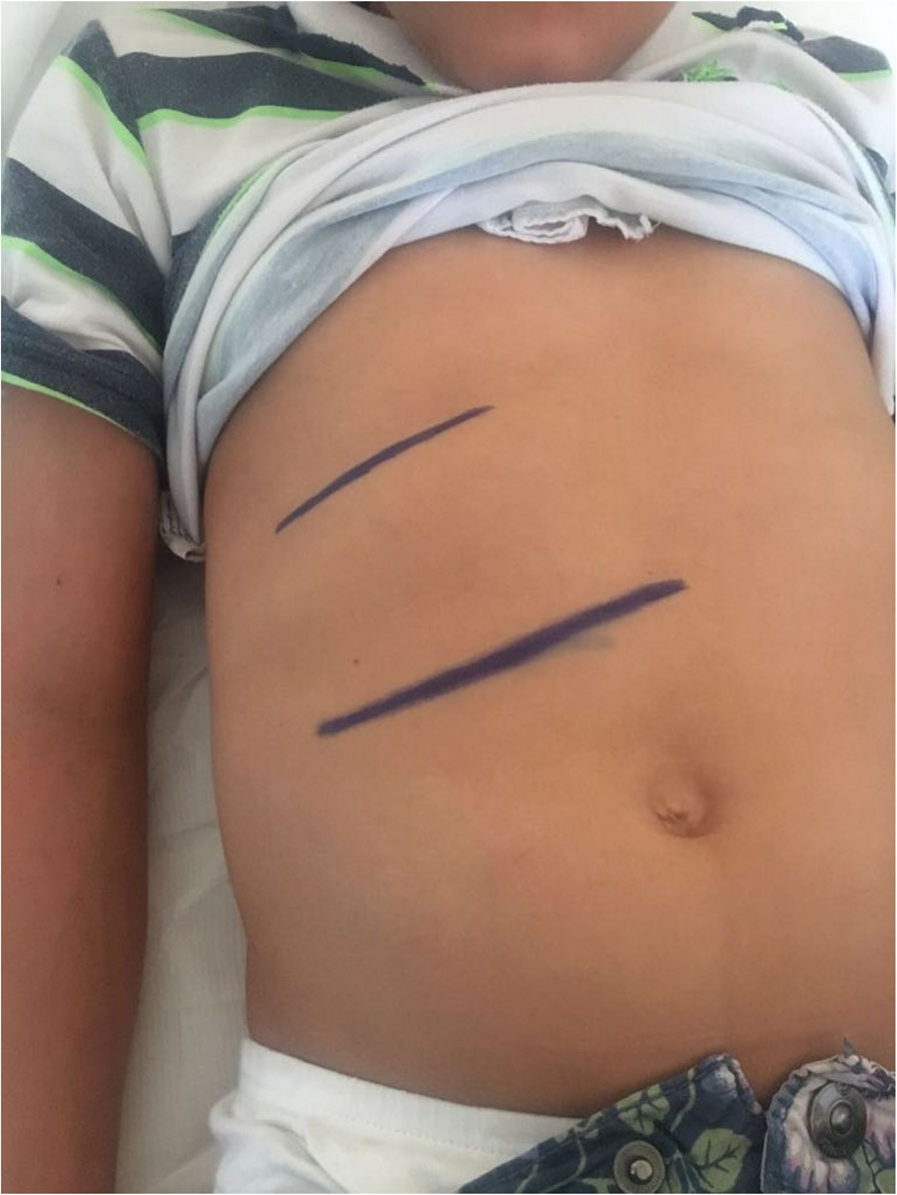
Reduction of hepatomegaly after intravenous continuous insulin treatment

**Fig. 4:**
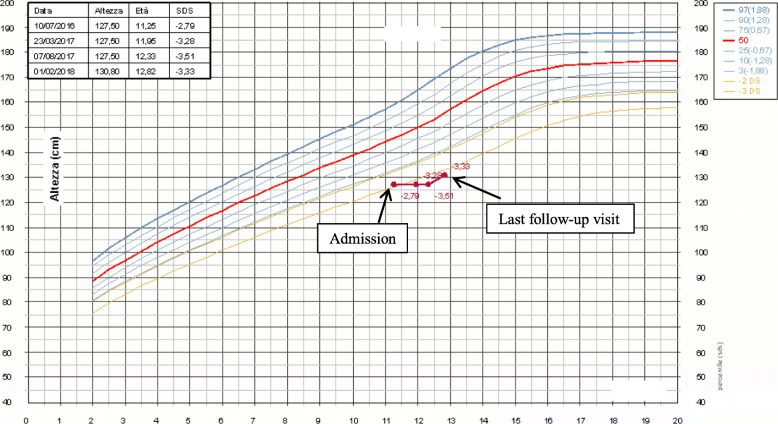
Patient’s growth chart since the admission to the last follow-up visit

At the three-month follow-up visit, he presented a poor glyco-metabolic control, glycated haemoglobin 124 mmol/l and extreme glycaemic variability. On physical examination, he had a more severe hepatomegaly. Laboratory tests showed total cholesterol 450 mg/dl, triglycerides 995 mg/dl, ALT 807 UI/L, AST 694 UI/L. Therefore, he was hospitalized and intravenous continuous insulin treatment was practiced for normalization of aminotransferases and achievement of good glycaemic control, reached after eight days. At the last follow-up visit the patient maintained a good glycemic control such as demonstrated by the value of glycated hemoglobin (55 mmol/l). The improvement of glycol-metabolic control lead to a complete remission of biochemical, clinical signs and complete resolution of hepatomegaly (Fig. 3). Despite to the regression of the liver disease, his stature remained < 3° centile and his growth velocity had an initial improvement only for the last months of clinical observation (Fig. 4).

## Discussion and conclusions

In T1D patients with poor glycaemic control, two events are usually present, promoting hepatic glycogen deposition: hyperglycaemia (as pointed out by increased blood glucose level and glycated haemoglobin) and consequent large amount of insulin (as demonstrated by elevated insulin dose as IU/kg of body weight/day). In hyperglycaemia, glucose passively enters the hepatocytes by the insulin-independent membrane glucose transporter GLUT2, and it is rapidly phosphorylated with inhibition of its release from hepatocytes [25]. Glucokinase convert the glucose into glucose-6-phosphate, with subsequent trapping in the hepatocyte. Then, an increased insulin administration promotes the polymerization of glucose-6-phosphate in glycogen by glycogen synthase, driving the large amount of glycogen synthesis in the presence of high cytoplasmic glucose concentrations [26]. Therefore, glycogen is trapped within the hepatocytes as a result of a combination of both hyperglycaemia and over-insulinization. The consequent liver damage become evident with the blood release of aminotransferases. Recently MacDonald et al. hypothesized that a heterozygous mutation found in a subunit of the liver glycogen phosphorylase kinase enzyme complex, combined with the extreme hyperglycaemia acted together to inhibit glycogenolysis and to cause massive glycogen accumulation in the liver cells. This large enzyme complex activates glycogen phosphorylase, which is the enzyme that catalyzes the first step in glycogen breakdown in the liver. In particularly, they found a heterozygous G ➔ A mutation in exon 9 of the PHKG2 gene, encoding the ɣ-subunit of phosphorylase kinase, which causes an arginine-to-glutamine (R309Q) amino acid change within domain N of PHKG2 [27].

To date MS during childhood is uncommon especially with the advent of new insulin analogues and intensive insulin regimens. However, there have recently been increasing numbers of case reports describing glycogen-induced hepatomegaly in T1D patients without other components of MS [28, 29]. More frequently, patients affected are teenager or young adults [5]. It is well known as adolescence is a critical period for T1D patients. In addition to experiencing the same challenges of his diabetic peers, our patient belonged to a family of immigrants with low socioeconomic level and his parents were unable to manage his disease. The concomitance of all these factors led to a brittle glycaemic control and the recurrence of DKA episodes, which are believed to be the principal risk factors in the development of HG.

Differential diagnosis include other causes of hepatopathy: hemochromatosis, infectious and autoimmune disease, metabolic, non-alcoholic fatty liver disease, obstructive and oncologic causes. Blood investigations and liver tests are fundamental to exclude most of these conditions. Some authors emphasized the elevation of lactate serum as a possible biomarker of HG in young diabetic patients [30, 31]. The ultrasonographic examination of the liver is a simple and useful procedure to have information about the dimension and the characteristics of the liver tissue [25], but it is not gold standard exam. Radiological imaging studies such as computed tomography and magnetic resonance imaging in establishing the diagnosis of HG could depend on the interval changes in the liver density, but to date their sensibility and specificity are not established for this condition [32]. Murata et al. reported the potential use of gradient-dual-echo magnetic resonance imaging sequence of the liver as a non-invasive and useful tool for diagnosis of HG by distinguishing from non-alcoholic fatty liver disease [33]. The gold standard examination to make diagnosis of HG is liver biopsy [34]. The main histological features of HG are marked glycogen accumulation leading to pale swollen hepatocyte, no or mild fatty change, no or minimal inflammation, no or minimal spotty lobular necrosis, and intact architecture with no significant fibrosis [28].

HG is completely reversible with a good metabolic control [35]. Continuous intravenous insulin infusion should be consider as an option when intensive insulin therapy and adequate nutritious diet are not sufficient to arrest the progression of liver disease and prevent further complications. A most recent systematic review hypothesized that HG could be diagnosed conservatively, based on medical history, physical examination, laboratory tests, imaging studies and response to treatment. These authors prompt the execution of liver biopsy only in case of doubt about the diagnosis or lack of clinical response [36].

MS became an uncommon condition since the emergence of intensive insulin treatment has allowed the achievement of ideal glycaemic targets in young diabetics. The rarity of the disease made sure that awareness of this clinical condition by clinicians, including specialists, is low [37]. We wish to highlight the recognition of key clinical signs, such as a brittle glycaemic control and hepatomegaly in T1D patients, to avoid that HG remained misdiagnosed. Recent reports described that the coexistence of some risk factors such as low socio-economic strata, adolescent age, high daily insulin dosage could prompt to a closer inspection. Further studies are required to demonstrate the role of genetic mechanism in the onset of this disease.

## Abbreviations

HG: Hepatic glycogenosis
T1D: Type 1 diabetes
MS: Mauriac’s syndrome
DKA: Diabetic ketoacidosis
